# Laser Desorption-Ion Mobility Spectrometry of Explosives for Forensic and Security Applications

**DOI:** 10.3390/molecules30010138

**Published:** 2025-01-01

**Authors:** Giorgio Felizzato, Martin Sabo, Matej Petrìk, Francesco Saverio Romolo

**Affiliations:** 1 Department of Law, University of Bergamo, Via Moroni 255, 24127 Bergamo, Italy; giorgio.felizzato@unibg.it; 2 Department of Drug Science and Technology, University of Turin, Via Giuria 9, 10125 Torino, Italy; 3 MaSa Tech, s.r.o., Sadová 3018/10, 916 01 Stará Turá, Slovakia; martin.sabo@masatech.sk (M.S.);; 4 Faculty of Informatics and Information Technologies, Slovak University of Technology in Bratislava, Ilkovičova 2, Bratislava 4, 842 16 Bratislava, Slovakia

**Keywords:** IMS, chemometrics, multivariate data analysis, data pretreatment, explosives, crime scene investigation, forensic, environmental and security applications

## Abstract

Background: The detection of explosives in crime scene investigations is critical for forensic science. This study explores the application of laser desorption (LD) ion mobility spectrometry (IMS) as a novel method for this purpose utilising a new IMS prototype developed by MaSaTECH. Methods: The LD sampling technique employs a laser diode module to vaporise explosive traces on surfaces, allowing immediate analysis by IMS without sample preparation. Chemometric approaches, including multivariate data analysis, were utilised for data processing and interpretation, including pre-processing of raw IMS plasmagrams and various pattern recognition techniques, such as linear discriminant analysis (LDA) and support vector machines (SVMs). Results: The IMS prototype was validated through experiments with pure explosives (TNT, RDX, PETN) and explosive products (SEMTEX 1A, C4) on different materials. The study found that the pre-processing method significantly impacts classification accuracy, with the PCA-LDA model demonstrating the best performance for real-world applications. Conclusions: The LD-IMS prototype, coupled with effective chemometric techniques, presents a promising methodology for the detection of explosives in forensic investigations, enhancing the reliability of field applications.

## 1. Introduction

Ion mobility spectrometry (IMS) is an analytical technique that uses an electric field and a supporting gas flow to separate analytes’ gas-phase ions according to their sizes and charge ratios [1]. In IMS, analyte ions are commonly generated in a reaction chamber through chemical reactions between sample molecules and reactant ions, particularly in ionisation methods like atmospheric pressure chemical ionisation (APCI) or corona discharge. A chain reaction can occur, leading toward the formation of several ion species [2]. The extent of fragmentation depends on various factors, such as temperature. At elevated temperatures and low moisture, high fragmentation is likely to occur [3].

IMS can provide three different outputs in order to characterise analytes’ ions: drift time *t_d_* (the arrival time at the detector of the analytes), ion mobility K, and reduced mobility K_0_.

$$K=\frac{v}{E}=\frac{L^{2}}{Vt_{d}}\;\left[1.0\right]$$

$$K_{0}=K\frac{P}{760}\frac{273}{T}\;\left[1.1\right]$$

*v* is the total voltage; *E* is the electric field; *t_d_* is the drift time; *L* is the tube region length; *P* is the pressure of the drift region; and *T* is the temperature of the drift gas.

Drift time and ion mobility are correlated to the instrument parameters and the experimental conditions. As a result, they have only in-house utility. Reduced mobility and cross-section values do not change among different instrument platforms [4]. Reduced mobility values are constant for a given compound in a specific drift gas and serve as a qualitative indicator of an ion’s features. However, reduced mobility can change over time due to instrumental parameters, such as inhomogeneities in temperature and the electric field, which are often poorly characterised. Moreover, it is also affected by pressure and environmental moisture [5]. Furthermore, the presence of interferences could complicate analyte identification. Post-run data pre-processing and analysis are often performed in order to overcome the aforementioned issues [6], offering various advantages including quick ion separation, effortless compatibility with pre-separation and gas-phase detection methods, enhanced selectivity, and the potential for miniaturisation.

IMS has been employed in many fields of forensic analysis, such as chemical weapons [7,8,9], explosives [10], and illicit drugs [11,12].

An efficient sampling technique is an important task for the detection of low-volatile explosives. Thermal desorption is a reliable technique traditionally used for IMS instruments [13]. With this technique, the investigated sample is transferred into a thermal desorption unit, where evaporation of the sample occurs. For surface analysis, wipe pads are usually used to collect samples from an investigated surface [14,15]. Laser desorption (LD) is an effective sampling technique for IMS and was introduced for the first time by Huang et al. in 1987 [16]. In his study, the authors used a relatively expensive and bulky Nd:YAG laser, possibly limiting the wider use of this desorption approach. The LD system studied in this research is based on a laser diode module and was described for the first time in 2014 by Sabo et al., who demonstrated the high efficiency of LD sampling for IMS [17]. This approach allows analysis of energetic material without any further sample treatment.

An effective sampling technique is not sufficient; it is also important to use efficient and appropriate data processing, which can improve the overall analytical capability of IMS instruments. Modern analytical instruments provide sizeable quantities of multivariate data. Chemometrics is employed in order to perform reliable and structured processing, analysis, and interpretation of analytical data. Furthermore, within complex criminal cases, chemometrics can provide additional information by analysing extensive sets of experimental data for police tactical or intelligence tasks [18].

In this study, we aimed to demonstrate the effectiveness of the combination of LD-IMS sampling with chemometrics data analysis for the detection of explosives during crime scene investigations.

## 2. Materials and Methods

### 2.1. Instruments

**Figure 1:**
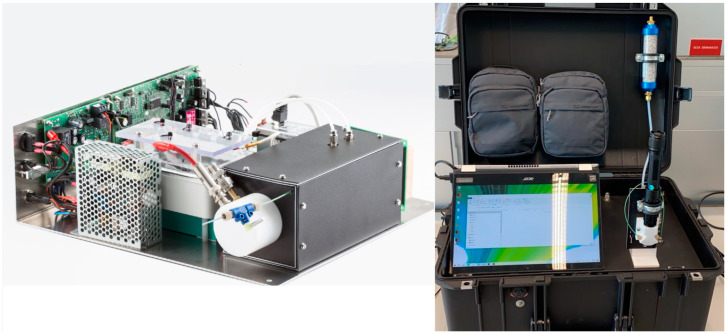
The IMS MaSaTECH prototype without the protective case (**on the left**) and the LD-IMS prototype in the protective case (**on the right**).

In this article, a new IMS prototype developed by MaSaTECH (www.masatech.eu) was tested within the RISEN project (www.risen-h2020.eu) for the detection of explosives during crime scene investigations. The IMS instrument is based on the Original Equipment Manufacturer–Advanced Ion Mobility Spectrometer (OEM-AIMS by MaSaTECH, Stará Turá, Slovakia) integrated into a protective case together with a long-lifetime battery (6 h of work) and a small membrane pump (Pfeiffer Vacuum, Korneuburg, Austria) see Figure 1 below. The small membrane pump keeps the IMS drift tube at sub-atmospheric pressure (600 mbar), which allows continuous aspiration of environmental air. The LD module, with a wavelength of 532 nm (green) and a power of 1 Watt, was placed in front of the sniffing capillary. The focused laser beam promotes sample evaporation, and then the sample is immediately aspirated and analysed by IMS.

The IMS instrument was used in negative-polarity mode. Hexachloroethane (CAS 67-72-1) was used as a chemical dopant for modification of reactant ions in order to achieve better sensitivity of IMS for explosive detection [19].

### 2.2. Target Compounds

**Figure 2:**
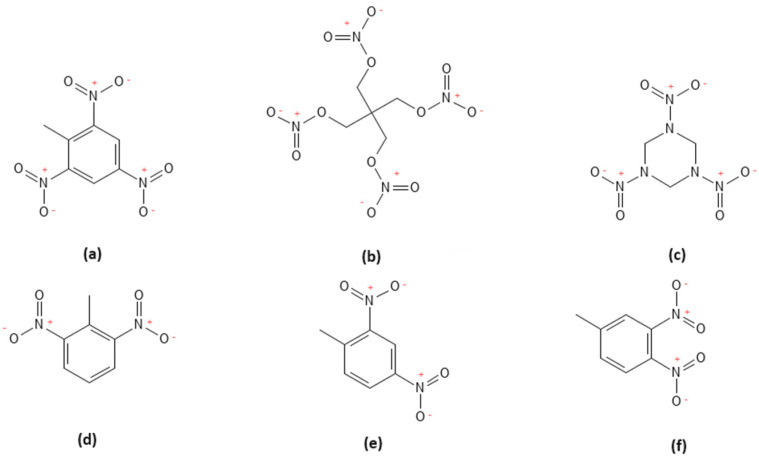
Molecular structures of (**a**) TNT, (**b**) PETN, (**c**) RDX, (**d**) 2,6 DNT, (**e**) 2,4 DNT, and (**f**) 3,4 DNT.

In this study, a selection of explosives was analysed, including TNT (2,4,6-trinitrotoluene), RDX (1,3,5-trinitrotriazinane), and PETN (pentaerythritol tetranitrate), which are commonly used in explosive formulations [20]. Notably, the plastic explosive product SEMTEX, tested during this study, is a mixture of two compounds: PETN and RDX. There are two different sorts of SEMTEX, which can be discriminated based on the ratio between PETN and RDX: 1A (PETN/RDX 0.94/0.06) and H (PETN/RDX 0.5/0.5) [21]. The plastic explosive product C4, which is composed of 91% RDX as an explosive filler, while the remaining part is composed of a plasticiser and a binder [22], was also tested. The molecular structures of the tested explosives are shown in Figure 2.

TNT, RDX, PETN, 2,6 DNT, 2,4 DNT, 3,4 DNT, C4, and SEMTEX 1A were obtained from the Slovak Police Academy (Bratislava, Slovakia). Moreover, methanol, acetone from Slavus s.r.o. (Bratislava, Slovakia), and distillate water were used as solvents.

The explosive stock solutions were prepared by dissolving the compounds in methanol to reach the final concentration of 1 mg/mL. By means of Eppendorf micropipettes (Hamburg, Germany), 5 microliters of solution was settled onto stainless steel, drywall, aluminium, ceramic, and PVC, generating different spots. Each one had a different surface concentration on the surface due to their different spreading patterns. Spots were heated by the laser beam, included in the IMS prototype, to promote analyte evaporation.

Each analyte was analysed in 12 replicates, and the analytical method was validated for both repeatability and within-laboratory reproducibility. Repeatability assesses the consistency of results obtained independently on the same sample under identical conditions—the same laboratory, operator, and equipment—within a short time frame. This was calculated based on six independent measurements, with confirmation on the following day. Within-laboratory reproducibility, on the other hand, evaluates the agreement of results from independent analyses on the same sample under varying conditions, such as different operators, reagent batches, solvents, and room temperatures. Reproducibility was determined from six measurements conducted by two operators across different environmental conditions.

Multivariate data analysis was carried out using Python code (v. 3.13.1) within a Jupyter Notebook environment. The following packages were used in the modules: NumPy [23], Pandas [24], Matplot library [25], Plotly [26], and Scikit-Learn [27]. Jupyter Notebooks are freely available at the following link: https://github.com/article-git/IMS_explosives_detection_by_machinelearning (accessed on 1 November 2024).

## 3. Results and Discussion

### 3.1. Validation Results

**Figure 3:**
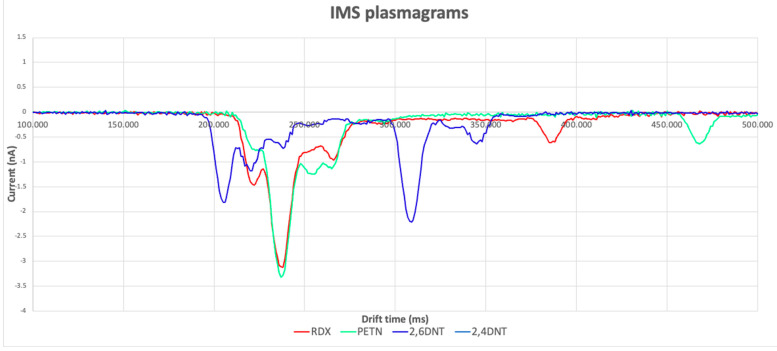
IMS plasmagram obtained by analysing RDX, PETN, 2,6-DNT, and 2,4-DNT.

The results of our experiments are shown as plasmagrams, as in Figure 3.

The analytical method underwent a validation process following the European Net-work of Forensic Science Institute guidelines and the Commission Implementing Regulation (EU) 2021/808 based on a validation plan for qualitative methods [28,29]. For the qualitative method developed in this study, the repeatability and within-laboratory reproducibility were taken into account.

The validation results for the repeatability and within-laboratory reproducibility of each compound analysed are shown in Table 1, with only the reduced mobility K_0_ of the main peak of each analyte considered. Table 1 presents the repeatability data, including the mean peak position, standard deviation, and percentage standard deviation. The low standard deviation percentages—ranging from 0.152% for TNT to 0.372% for 2,6-DNT—demonstrate high precision for measurements taken under identical conditions within a single laboratory. Notably, TNT and 2,4-DNT displayed the lowest variability, with standard deviation percentages of 0.152% and 0.204%, respectively, suggesting that these compounds can be consistently measured with minimal fluctuation. Moreover, Table 1 provides the within-laboratory reproducibility results, which show the variability across measurements taken under different operators and environmental conditions. The reproducibility percentages remained within a reliable range, with standard deviations between 0.159% for TNT and 0.293% for PETN. Compounds like 2,6-DNT and 3,4-DNT showed slightly higher variability, with standard deviation percentages of 0.286% and 0.289%.

The validation results highlight the robustness of the method across different analytical setups within the same laboratory environment. The low levels of variation, both under the same operator conditions and across different setups, underscore the method’s reliability for detecting and analysing these explosive compounds.

Validation of the IMS sensor was carried out by employing the experimental conditions reported in Table 2.

### 3.2. IMS Multivariate Data Analysis

In this study, various chemometric methodologies were developed to identify patterns within the spectral data, enabling the differentiation of samples that might otherwise appear similar based solely on reduced mobility or drift times. These approaches enhance the ability to distinguish subtle spectral features, improving the accuracy and reliability of sample classification in complex datasets.

**Figure 4:**
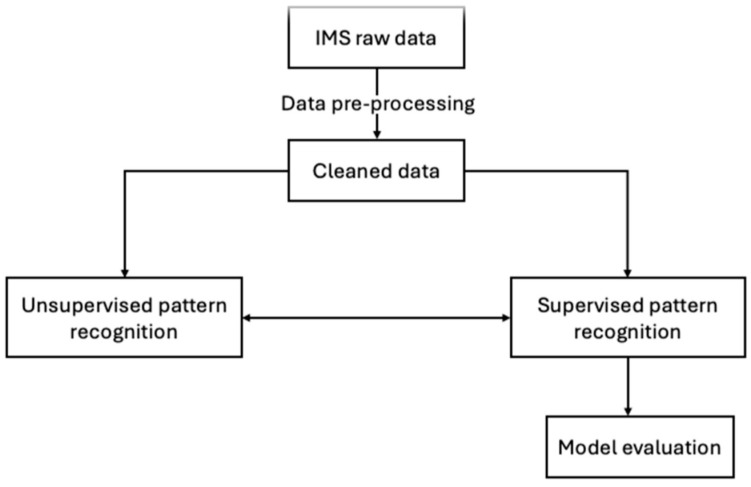
Chemometrics workflow for the IMS data.

IMS spectra were treated using the following stepwise chemometrics approach (Figure 4):

- Pre-processing of raw data;
- Unsupervised pattern recognition;
- Supervised pattern recognition;
- Model assessment by means of cross-validation.

A Design of Experiment (DoE) approach was used to identify the best combination of pre-processing techniques and determine which factors influence the model’s response by using the accuracy of the final chemometrics model. One advantage of DoE-based approaches, particularly with a full factorial design, is the exploration of all possible pre-processing combinations. A full factorial design of 2 levels (low level, “−”; high level, “+”) and 3 factors was employed, yielding 2^3^ = 8 for the number of experiments required for each classification model. During this study, the following methods were employed: standard normal variate (SNV), Savitzky–Golay smoothing, and autoscaling. The combinations involving SNV and *autoscaling* were not taken into account, leading to a reduction in the total pre-processing combinations to six. The low level for a factor means that no pre-processing was performed, while a high level indicates that pre-processing was applied. IMS data pre-processing consists of steps such as denoising, baseline correction, data scaling, and normalisation. The main goal of denoising techniques is to discriminate noise signals as high-frequency variations from analyte signals as low-frequency variations. The final design matrix with three factors is shown in Table 3 [30,31].

Unsupervised pattern recognition was carried out as the first step of the data analysis to allow the identification of the key features within the dataset. We carried out principal component analysis (PCA) to explore the data acquired by the IMS prototype using four PCs (reaching a cumulative variance explained of 87%). Subgroups or clusters within the principal component space were evaluated, as well as possible outliers. Each pre-processing combination was evaluated in terms of class overlapping and the number of outliers. For this purpose, Hotelling-T^2^ and Q residuals were used in this article by applying a 95% confidence level.

**Figure 5:**
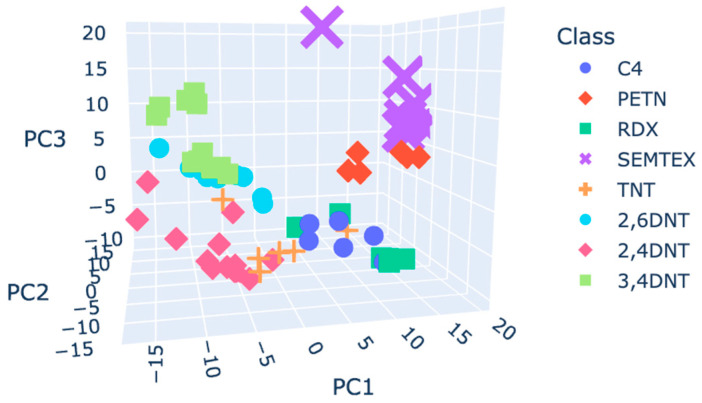
Score plot coloured by class using the autoscaling pre-processed plasmagrams.

Using the raw plasmagrams, the first two PCs effectively differentiated DNT isomers, but other classes overlapped, notably, C4 and RDX. TNT data points did not form their own cluster but spread in the space. For the smoothed plasmagrams, different parameters of a Savitzky–Golay filter were evaluated. When using a derivate higher than the order 0, the number of outliers increases. No differences were found when using different combinations of window size and polyorder. A window size of 15 and a polyorder of 6 were chosen to compute the principal component analysis. The clusters’ shapes, the classes’ positions in the principal component space, and the numbers of outliers were similar in the smoothed plasmagrams compared to the raw plasmagrams. SNV maintained good separation of DNT isomers and allowed better separation of SEMTEX and PETN. A higher number of outliers was recognised versus the number obtained using the raw data. Smoothing before the SNV did not significantly change the final PCA results. Autoscaling improved class separation, forming distinct clusters for each class except for C4 and RDX, which remained closely positioned. Figure 5 shows the PCA 3D plot for the autoscaled data. The combination of smoothing plus autoscaling resulted in worse separation.

**Figure 6:**
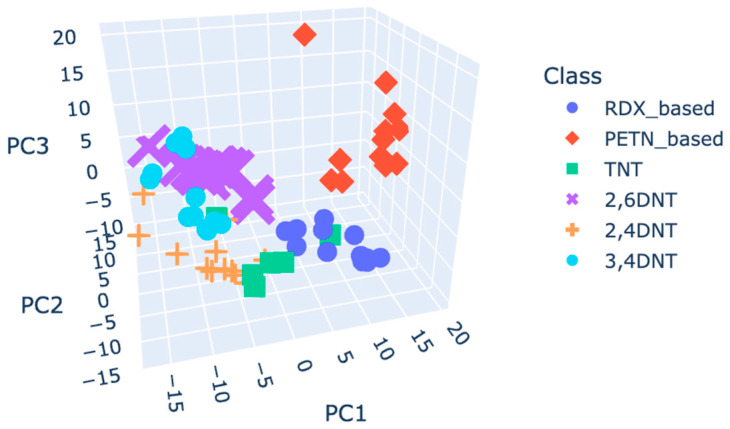
Score plot of grouped classes, coloured by class, using the autoscaling pre-processed plasmagrams.

To address the overlapping issues, classes were reorganised according to their active explosive compounds: C4 and RDX were merged into a unified category named “RDX based”, and SEMTEX 1A and PETN were combined into a category named “PETN based”. Figure 6 shows the corresponding score plot for the autoscaling pre-processed plasmagrams.

The leading classification method for IMS data is often linear discriminant analysis (LDA). This analysis can be conducted either on a cleaned dataset or following PCA analysis with a chosen set of principal components, known as the PCA-LDA approach [6,32,33]. We computed the LDA after the PCA by using the scores as input data to overcome the correlation of drift times (variables). Six different pre-processing methods were applied to the raw data before conducting the PCA, and the most effective one was determined based on the accuracy of the LDA classification model. Considering that PCA involved four principal components, the LDA was consequently computed with three latent variables. Model evaluation was conducted by splitting the dataset into the training set and the evaluation set; 70% of the samples were used in order to set up the model, and the remaining 30% was used to assess the model itself. Table 4 shows the accuracy of the model for different combinations of the aforementioned pre-processing techniques.

**Figure 7:**
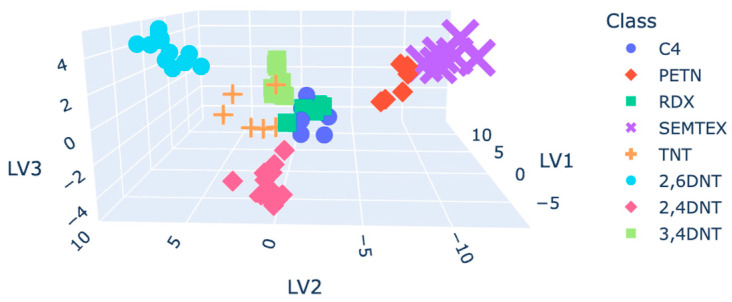
D plot coloured by class for the PCA-LDA.

Autoscaled plasmagrams, as expected, tended to reflect the highest model accuracy. Figure 7 shows the data points within the 3D latent variable plot. C4 and RDX were not discriminated from each other, affecting the accuracy of the final model.

**Figure 8:**
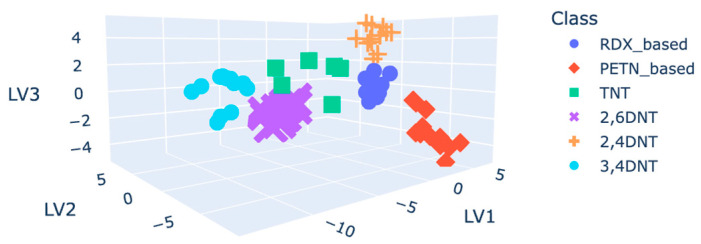
D plot of grouped classes, coloured by grouped class, for the PCA-LDA.

The PCA-LDA was also computed by using the grouped dataset. The model was executed by using the autoscaled plasmagrams. The resulting 3D plot is shown in Figure 8, where all the classes are well separated from each other.

By means of cross-validation, an accuracy of 100% was obtained. Five folds were used for the cross-validation. This number was considered appropriate to ensure that both the training and test sets had sufficient samples to produce reliable results. The use of more folds would leave the test sets too small, making the evaluation less reliable. On the other hand, using fewer folds would leave the training sets too small, leading to poorly trained models. The accuracy value obtained for the PCA-LDA model was expected to decrease considering more experimental data.

Partial least square–discriminant analysis (PLS-DA) [34] was computed using six different pre-processing methods, as previously employed. The optimal approach was determined by computing the model’s accuracy (results shown in Table 4). The accuracy values were numerically very close to each other and notably lower than the PCA-LDA values.

Logistic regression (LR) [35,36] was computed after the PCA using the obtained scores as variables for setting up the classification model in order to overcome the correlation of drift times. To ensure a comprehensive data analysis, the PCA was computed using six distinct pre-processing methods, as previously described. The efficiency of each approach was assessed based on the accuracy of the final classification model. Two solvers, “liblinear” and “Newton-cg”, were selected for their roles as optimisation algorithms to determine the coefficients that optimised the model’s fit to the data. The solver “liblinear” uses a coordinate descent (CD) algorithm where the optimisation problem is decomposed in a “one-vs-rest” technique, so independent binary classifiers are trained for all classes. The coordinate descent (CD) is an optimisation algorithm that finds the minimum of the function along the coordinate direction separately. It can deal with a wide range of different training data and is used as a default solver due to its robustness. “Newton-cg” is useful for high-dimensional datasets. It considers all classes simultaneously, learning a true multinomial logistic regression model. This approach, unlike the default “one-vs-rest” setting, was expected to provide better-calibrated probability estimates [27].

Both the solvers were evaluated by computing the confusion matrix on the evaluation set. The process was carried out for each pre-processing technique involved in the data analysis. The model accuracy values are shown in Table 4 below.

The “Newton-cg” solver coupled with Savitzky–Golay smoothing or the standard normal variate provided the highest value for model accuracy.

*Support vector machines (SVMs)* [37,38] were computed by using four different kernels: linear, poly (polynomial), rbf (radial basis function), and sigmoid. Each kernel was evaluated in terms of model accuracy using plasmagrams that were pre-processed by employing six different methodologies. The assessment was conducted using the evaluation set. The accuracy values are shown in Table 4.

The autoscaled plasmagram, previously smoothed, provided the highest value for accuracy for the linear, radial basis function, and sigmoid kernels. Moreover, the use of raw data resulted in similar accuracy using the polynomial kernel.

## 4. Conclusions

The effectiveness of the new prototype of laser desorption IMS developed by MaSaTECH for the detection of explosive traces distributed on surfaces typically found during crime scene investigations has been explored and demonstrated. In addition, the study included a chemometrics approach for data analysis in order to enhance the accuracy and reliability of detection.

The chemometrics workflow involved various steps, including pre-processing of raw data, unsupervised and supervised pattern recognition, and model assessment through cross-validation. The pre-processing method, including denoising, baseline correction, and scaling, played a crucial role in enhancing the quality of the data and the quality of the models.

Linear discriminant analysis (LDA) stood out as the most effective classification method using the autoscaled plasmagrams. An accurate classification model was obtained by grouping classes of explosives based on their active compounds. Furthermore, other classification models such as partial least squares discriminant analysis (PLS-DA), logistic regression (LR), and support vector machines (SVMs) were evaluated. The PLS-DA model accuracy obtained was numerically lower than that of the PCA-LDA model. Logistic regression was applied following principal component analysis (PCA) to address the correlation among drift times. The use of the “Newton-cg” kernel for setting up the model provided an accuracy of 90% when the data were pre-processed by the SNV. High accuracy was achieved when autoscaled plasmagrams were employed with linear, radial basis function, and sigmoid kernels.

In sum, the IMS prototype developed by MaSaTECH coupled with the chemometrics approach described in this article was shown to be a promising methodology for the detection of explosives during crime scene investigations.

## Figures and Tables

**Table 1:** Repeatability and within-laboratory reproducibility results for the IMS prototype.

	Repeatability			Within-Laboratory Reproducibility		
Compound	Mean Peak Position (K_0_)	Standard Deviation	RDS%	Mean Peak Position (K_0_)	Standard Deviation	RDS%
C4	1.39	0.00268	0.193	1.39	0.00385	0.278
PETN	1.16	0.00297	0.256	1.16	0.00339	0.292
RDX	1.39	0.00257	0.184	1.39	0.00340	0.244
SEMTEX	1.16	0.00259	0.223	1.16	0.00245	0.210
TNT	1.45	0.00221	0.152	1.45	0.00230	0.159
2-4-DNT	1.36	0.00277	0.204	1.36	0.00277	0.204
2-6-DNT	1.48	0.00549	0.372	1.48	0.00422	0.286
3-4-DNT	1.35	0.0028	0.209	1.36	0.00391	0.289

**Table 2:** IMS experimental conditions.

Experimental Condition	
Drift field intensity [V/cm]	509.314
Pressure [mbar]	602.339
Temperature [k]	335.5313
Drift tube length [cm]	11.14

**Table 3:** Full factorial design of the pre-processing methods.

Experiments	Smoothing	Standard Normal Variate	Autoscaling
1	−	−	−
2	+	−	−
3	−	+	−
4	−	−	+
5	+	+	−
6	+	−	+
7	−	+	+
8	+	+	+

**Table 4:** Full factorial design for machine learning model assessment.

		Model Accuracy								
			PCA-LDA	PLS-DA	PCA-LR		Support Vector Machines			
Plasmagram Pre-Processing					“Newton-cg” Solver	“Liblinear” Solver	Linear Kernel	Poly Kernel	Rbf Kernel	Sigmoid Kernel
Experiments	Smoothing	Standard Normal Variate								
1	−	−	0.90	0.55	0.75	0.75	0.85	0.95	0.85	0.60
2	+	−	0.90	0.60	0.90	0.80	0.70	0.75	0.80	0.60
3	−	+	0.85	0.55	0.90	0.75	0.80	0.80	0.75	0.60
4	−	−	0.95	0.50	0.85	0.75	0.90	0.20	0.75	0.90
5	+	+	0.80	0.50	0.80	0.80	0.85	0.85	0.85	0.65
6	+	−	0.90	0.50	0.80	0.65	0.95	0.20	0.95	0.95

## Data Availability

Data are contained within the article.
